# Immunogenic Properties of a Novel Hepatitis A Vaccine Candidate Based on a Fast-Growing Viral Strain

**DOI:** 10.3390/vaccines13050446

**Published:** 2025-04-23

**Authors:** Maria Isabel Costafreda, Malén Massot-Cladera, Gemma Chavarria-Miró, Alba Arrebola, Àngels Franch-Masferrer, Maria J. Rodríguez-Lagunas, Adán Martínez-Velázquez, Albert Blanco, Albert Bosch, Susana Guix, Margarida Castell, Rosa Maria Pintó

**Affiliations:** 1 Enteric Virus Laboratory, Department of Genetics, Microbiology and Statistics, School of Biology, University of Barcelona, 08028 Barcelona, Spain; gemmach95@gmail.com (G.C.-M.); aarrebola@ub.edu (A.A.); adanmartinez@ub.edu (A.M.-V.); ablancoortiz@ub.edu (A.B.); abosch@ub.edu (A.B.); susanaguix@ub.edu (S.G.); rpinto@ub.edu (R.M.P.); 2 Institute of Nutrition and Food Safety (INSA), University of Barcelona, Santa Coloma de Gramenet, 08921 Barcelona, Spain; malen.massot@ub.edu (M.M.-C.); angelsfranch@ub.edu (À.F.-M.); mjrodriguez@ub.edu (M.J.R.-L.); margaridacastell@ub.edu (M.C.); 3 Centro de Investigación Biomédica en Red de Enfermedades Hepáticas y Digestivas (CIBERehd), Instituto de Salud Carlos III, 28029 Madrid, Spain; 4 Autoimmunity, Immunonutrition and Tolerance Group, Department of Biochemistry and Physiology, Faculty of Pharmacy and Food Science, University of Barcelona, 08028 Barcelona, Spain; 5 Centro de Investigación Biomédica en Red de Fisiopatología de la Obesidad y la Nutrición (CIBEROBN), Instituto de Salud Carlos III, 28029 Madrid, Spain

**Keywords:** hepatitis A virus, HAV, immunization, immune protection, cost-effectiveness

## Abstract

Background/Objectives: Hepatitis A virus (HAV) yearly causes over 150 million new infections and around 40,000 deaths. Current vaccines are based on strains that grow poorly in cell culture, leading to high production costs and limited availability. This study aimed to compare the immunogenic properties of a novel HAV vaccine candidate based on the fast-growing HM175-HP strain with those of the parental slow-growing HM175-L0 strain, which derives from the cytopathic HM175 strain, like the prototype strain used in certain existing vaccines. Methods: The humoral and cellular immune response elicited by either HM175-HP or HM175-L0 vaccines was assessed in female BALB/c mice. Results: Both HM175-HP and HM175-L0 vaccines induced comparable levels of anti-HAV IgG, as well as similar numbers of antibody-secreting cells and cellular proliferation rates in immunized mice. Importantly, anti-HAV antibodies developed by HM175-HP-immunized mice were able to neutralize the HM175-L0 strain. In addition, both vaccines induced anti-HAV IgG1 antibodies, which are associated with Th2 immune response, but the HM175-HP vaccine showed a tendency to produce a greater IgG2a response, suggesting that it might elicit a higher Th1 response, which is of utmost importance for host defense against viruses. Conclusions: Our findings indicated that the fast-growing HM175-HP strain has similar immunogenic properties to the vaccine prototype-like HM175-L0, making it a promising candidate to reduce the elevated costs and time-consuming procedures of producing the current HAV vaccines. The novel HM175-HP-based vaccine would therefore facilitate mass vaccination programs and prevent vaccine shortages.

## 1. Introduction

Hepatitis A is an infectious liver disease caused by the hepatitis A virus (HAV). The incidence of hepatitis A varies widely across countries and strongly correlates with socioeconomic indicators, with the highest endemicity observed in low-income and lower-middle-income countries, according to World Health Organization (WHO) data [1]. Despite the existence of effective vaccines, over 150 million new cases are reported yearly. Most of them occur in highly endemic areas where people get infected during childhood, with infections normally asymptomatic and providing life-long immunity. However, the severity of the disease can increase with age, as well as other host factors, including immune status and pre-existing liver conditions [2]. As a result, HAV infections cause over 2M disability-adjusted life years and around 40,000 deaths yearly [1,3]. Hepatitis A outbreaks, mainly related to contaminated food and water, do occur in regions with very low or low hepatitis A endemicity due to the high proportion of susceptible adults who can develop clinically significant hepatitis A. Unusual increases in cases amongst men-having-sex-with-men (MSM) have also been recently documented, likely caused by sexual transmission, particularly oral–anal sexual contact. Consequently, the WHO advises vaccination for individuals from low-endemicity areas who are at high risk of HAV exposure or severe hepatitis A, including MSM, refugees, homeless, individuals who travel to highly endemic areas, and those with chronic liver disease or acquired immunodeficiency syndrome [3]. In addition, due to the increased incidence of hepatitis A in middle-income countries transitioning from high to intermediate endemicity, the WHO also recommends universal childhood vaccination in these areas. However, only about 30 countries have implemented these vaccination programs, with the cost and feasibility being major barriers for their implementation. Indeed, 33% of countries with hepatitis A vaccine in their routine programs are using a single-dose schedule to reduce cost [4]. For the same economic reason, 60% of countries are using single-dose schedules for vaccination of risk groups [4], which includes a percent of HIV-positive individuals that should receive three doses of the HAV vaccine to induce a protective immune response [5,6].

Current inactivated hepatitis A vaccines are based on cell culture-adapted HAV strains, including HM175/18f (HAVRIX^®^), CR-326 (VAQTA^®^), GBM (AVAXIM^®^), TZ84 (HEALIVE), Lv-8 (Weisairuian), KRM003 (Aimugen), YN5 (VERAXIMIR), RG-SB (EPAXAL^®^). These strains grow poorly in cell culture and produce low antigen yields, which results in an extremely costly and time-consuming production that explains, at least in part, the elevated costs and unaffordability of mass vaccination, as well as vaccine shortages related to a higher demand to control hepatitis A outbreaks.

The HM175-HP clone of HAV is a fast-growing strain derived from the parental slow-growing strain HM175-L0, also known as HM175/43c variant [7]. Like the prototype HAV vaccine HM175/18f, the HM175-L0 was derived from the cytopathic HM175 strain; thus, they share a high antigenic similarity [8]. In turn, the HM175-HP strain was obtained through the adaptation of the vaccine prototype-like HM175-L0 to grow under actinomycin D (AMD)-mediated transcriptional shut-off [9,10], followed by competition experiments between populations adapted to moderate and high shut-off levels [7]. An exhaustive characterization of the HM175-HP clone revealed three mutations in the internal ribosome entry site (IRES), two amino acid changes at positions 123 and 134 of the VP1 and VP2 capsid proteins, respectively, and an altered codon usage, particularly in the VP1 coding region [7,9,10]. These modifications contribute to the fast-growing phenotype because they result in the following: (i) a more efficient IRES to direct translation compared with the inefficient one in the parental HAV strain [7,11,12]; (ii) a more accessible late domain in the VP2, which facilitates the interaction of the viral capsid with ALIX during the virus egress (Carcereny et al.); and (iii) a faster translation rate in the capsid coding region due to an optimized codon usage [13], compared to the slow translation rate resulting from the deviated codon usage with respect to the cell codon usage that presents the parental strain [14]. Given its fast-growing phenotype, the HM175-HP HAV strain is capable of producing higher antigen yields in less time [13], which could therefore allow for the reduction in the elevated costs and time-consuming procedures required for HAV vaccine production.

The aim of this study was to evaluate the immunogenic capacity of the HM175-HP strain and compare it with that of the parental HM175-L0 strain, which resembles the prototype of some of the current marketed vaccines. Our findings indicate that both strains have similar antigenic structures and induce similar humoral and cellular immune responses, suggesting that the HM175-HP strain is indeed an excellent candidate for a novel HAV vaccine.

## 2. Materials and Methods

### 2.1. Cells, Viruses, and Antibodies

FRhK-4 cells, an epithelial-like cell line derived from the kidney of a fetal rhesus monkey (*Macaca mulatta*) that supports the growth of cell culture-adapted HAV HM175 strain, were kindly provided by Prof. Bertram Flehmig, University of Tübingen, Germany. Cells were grown in Eagle’s minimum essential medium (EMEM, Sigma-Aldrich, St. Louis, MO, USA) containing 15% inactivated fetal bovine serum (FBS, Lonza, Verviers, Belgium), 2 mM L-glutamine (HyClone, Logan, UT, USA), and 1% penicillin-streptomycin (Gibco, Gran Island, NY, USA).

The previously characterized HM175-HP population [7] and its ancestor, the cytopathogenic HM175/43c HAV strain (HM175-L0; courtesy of Dr. Theresa Cromeans, Centers for Disease Control, Atlanta, GA, USA), were used throughout this study. Both were grown in FRhK-4 cells in the presence of maintenance medium consisting of EMEM with 2% inactivated FBS, 2 mM L-glutamine, and 1% penicillin-streptomycin. For comparing the antigenic properties of HM175-L0 and HM175-HP, both were grown in the presence or absence of 0.05 µg/mL of Actinomycin D (AMD, Sigma-Aldrich).

Human anti-HAV IgG and peroxidase-labeled human anti-HAV IgG, both derived from convalescent plasma and kindly provided by Biokit (Barcelona, Spain), were used to capture and detect HAV capsids by ELISA, respectively. Peroxidase-labeled goat anti-mouse IgG (Merck, Darmstadt, Germany) was used to detect mouse plasma-specific antibodies. Biotin-conjugated anti-mouse IgG1 and IgG2a (both from Merck) were used to detect specific IgG1 and IgG2a antibody isotypes, respectively. Biotin-conjugated anti-mouse IgG antibody (Merck) was used to detect antibody-secreting cells.

The Mouse Naïve/Memory T cell ID Panel (Biolegend, San Diego, CA, USA), consisting of APC/Cy7-conjugated anti-CD3 (clone 17A2), PerCP/Cy5.5-conjugated anti-CD4 (clone RM4-5), APC-conjugated anti-CD62L (clone MEL-14), and PE-conjugated anti-CD44 (clone IM7) was used to detect the different subpopulations of naïve and memory T cells. FITC-conjugated anti-CD8 (clon 53-6.7) and Brilliant Violet 421 anti-mouse IFN-γ (clon XMG1.2) (Biolegend) were used for CD8 subset and intracellular IFN-γ detection, respectively.

### 2.2. Virus Titration by Endpoint Dilution

Infectious viral titers were determined by an endpoint dilution assay in 96-well plates containing FRhK-4 cells and using 10-fold serial dilutions of viral samples in octuplicate wells. Cytopathic effect (CPE) was assessed at 10–13 days post-infection (p.i.) under the microscope, and infectious viral titers were calculated as tissue culture infectious doses 50% (TCID_50_) per mL.

For the neutralization assay, 10^5^ TCID_50_ of naked particles of HM175-L0 or HM175-HP in 0.1 mL of maintenance medium were incubated with 1/10 and 1/50 dilutions of plasma from mice immunized with HAV antigen or control antigen for 3 h at 37 °C. Neutralization mixtures were titrated by the endpoint dilution assays in FRhK-4 cells monolayers. The neutralization index of a plasma from a vaccinated mouse was calculated as the Log_10_ of the number of units per mL of plasma capable of neutralizing 50% of 1 × 10^5^ TCID_50_ units in 50% of the replicates (Log_10_ 50N_50_ U/mL).

### 2.3. Antigen Preparation and Vaccine Formulation

At day 7–10 p.i., cell culture supernatants were collected, centrifuged twice at 10,000× *g* for 30 min at 4 °C, ultracentrifuged at 100,000× *g* for 2 h at 4 °C in an SW32Ti rotor (Beckman Coulter, Miami, FL, USA), resuspended in PBS-15 mM HEPES and subjected to 3 freeze–thaw cycles. Then, HAV particles were stored at −80 °C until used.

For vaccine formulation, HAV particles at a concentration of 10^9^ TCID_50_/mL or control antigen (ovalbumin from chicken egg white, OVA, Merck) were UV-inactivated for 30 min and adjuvanted 1:1 with alum (Aluminum hydroxide; Imject^®^ Pierce, Rockford, IL, USA) 1 h prior to immunization.

### 2.4. Recognition by Human Convalescent Plasma

The recognition of HAV particles from HM175-L0 and HM175-HP populations by human convalescent plasma was assessed by ELISA. To do so, viral suspensions were incubated in the presence of 1% NP40 detergent (Merck) at 37 °C for 30 min, sonicated thrice at 60 W for 30 s, and subjected to 3 freeze–thaw cycles. Then, viral particles were captured by human anti-HAV IgG (0.05 µg/well) and detected with peroxidase-labeled human anti-HAV IgG diluted 1/40,000 in washing/blocking solution (1 × PBS containing 3 mg/mL BSA and 0.05% Tween 20). After developing the plates with 1-step Ultra TMB-ELISA (Thermo Scientific, Rockford, IL, USA), the reaction was stopped with 2 M H_2_SO_4_, and the color signal was measured at O.D. 450 nm using a microplate photometer (ASYS UVM340, Biochrom, Cambridge, UK).

### 2.5. Mice

Six-week-old male and female BALB/c (BALB/cOlaHsd) and female C57BL/6 (C57BL/6JolaHsd) mice were obtained from Envigo RMS (Sant Feliu De Codines, Spain). The animals were housed under controlled conditions of temperature (21 ± 2 °C) and humidity (50%) and under a light/dark cycle of 12 h in the Animal Experimentation Unit of the School of Pharmacy and Food Science at the University of Barcelona. After virus inoculation, animals were housed in Type II Biosecurity Conditions (biosecurity room in the same Animal Experimentation Unit under conditions of negative pressure). The animals were provided with food and water ad libitum.

All experimental procedures were conducted in accordance with the institutional guidelines for the Care and Use of Laboratory Animals and were approved by the Ethical Committee for Animal Experimentation of the University of Barcelona and the Catalonia Government (CEEA/UB ref. 149/19 and DAAM 10761, respectively), in full compliance with national legislation following the EU-Directive 2010/63/EU for the protection of animals used for scientific purposes [15]. The protocol outlined criteria for animal inclusion and exclusion. The approved euthanasia method consisted of diaphragm disruption in anesthetized animals. The necessary sample size was determined using the Appraising Project Office’s program from the Universidad Miguel Hernández de Elche (Alicante, Spain). This calculation, based on plasma-specific antibodies concentration as the variable, assumed no dropout rate and a two-sided type I error of 0.05. In addition, sample size was adjusted to the minimum needed to follow the University Ethical Committee guidelines.

### 2.6. Mouse Immunizations

Inbred BALB/c and C57BL/6 mice received 4 subcutaneous doses of 100 µL (50 µL of HM175-L0 or HM175-HP antigen containing 5 × 10^7^ TCID_50_ UV-inactivated combined with 50 µL of alum) every two weeks (i.e., on days 0, 14, 28, and 42 of the study). In addition, as a reference (REF) group, BALB/c mice were immunized with 100 µL of control antigen, consisting of 50 µL of OVA immunogen (400 µg/mL) and 50 µL of alum, following the same procedure. Blood samples were collected on days 0, 28, and 42 from the mandibular plexus, and on day 56 directly by heart puncture under isoflurane anesthesia (Ecuphar, Barcelona, Spain). Plasma samples were stored at −20 °C until the quantification of specific antibodies. In addition, on day 56, spleen samples were obtained following euthanasia.

### 2.7. Splenocyte Isolation

Spleen was removed aseptically and soaked in complete Roswell Park Memorial Institute medium (RPMI, Gibco) containing 10% FBS, 2 mM L-glutamine, 1% penicillin-streptomycin, and 0.25 µM 2β-mercaptoethanol (Merck).

Splenocytes were isolated in a biosafety cabinet by placing the spleen onto a 40 µm cell strainer (Thermo Fisher Scientific, Waltham, MA, USA) and smashing it using the plunger of a 2 mL syringe while maintaining cold conditions. The cell suspension was centrifuged at 538× *g* for 10 min at 4 °C, and erythrocytes were removed by osmotic lysis. Cell viability and concentration were assessed by an automated cell counter (Countess^TM^, Thermo Fisher Scientific).

### 2.8. Plasma Anti-HAV IgG, IgG1 and IgG2a Antibody Concentrations

An ELISA was performed to quantify anti-HM175-L0 and anti-HM175-HP IgG antibodies in plasma from immunized mice. In brief, ELISA plates were coated with human anti-HAV IgG in sodium carbonate/sodium bicarbonate buffer with pH 9.6 overnight at 4 °C and incubated with 50 µL (10^7^ TCID_50_/mL) of HM175-L0 or HM175-HP particles prepared as above (see Section 2.3) for 3 h at 37 °C. PBS buffer containing 3 mg/mL BSA and 0.05% Tween 20 was used as washing/blocking solution. Mouse plasma dilutions in PBS were incubated overnight at 4 °C. After washing 3 times, secondary peroxidase-conjugated goat anti-mouse IgG was incubated for 1 h at 37 °C. Afterwards, plates were washed and 1-step Ultra TMB-ELISA chromogen solution was added for developing the plates. The reaction was stopped with 2 M H_2_SO_4_, and the color signal was measured at O.D. 450 nm using a microplate photometer (ASYS UVM340). Results are expressed as the reciprocal of the highest plasma dilution providing a positive signal for each sample. The cutoff value for considering a positive signal was established as the mean + 3SD of the absorbance values obtained from plasma samples of the REF group (OVA-immunized animals).

To ascertain anti-HAV IgG1 and IgG2a isotypes, a similar ELISA was carried out as above. After plasma sample incubation, secondary biotin-conjugated anti-mouse IgG1 or IgG2a was added for 1 h at 37 °C and, after washing, extravidin-peroxidase (Merck) was incubated for 30 min at 37 °C. Plates were developed as stated before. Results are also expressed as the plasma titer considering the reciprocal of the highest plasma dilution providing a positive signal for each sample.

### 2.9. Quantification of Anti-HAV Antibody-Secreting Cells

The number of spleen cells secreting anti-HAV antibodies in BALB/c mice was enumerated by an enzyme-linked immunosorbent-spot (ELISPOT) assay. Briefly, 96-well hydrophobic PVDF membrane plates (0.45 µm; Thermo Fisher Scientific) were sensitized with anti-HAV convalescent human plasma overnight at 4 °C in a humid chamber, and later coated with HM175-HP virus (10^5^ TCID_50_, 100 µL/well) for 2 h at room temperature in a humid chamber. After washing with sterile PBS buffer, the plates were blocked with complete culture medium for 1 h at 37 °C. Serial dilutions of spleen lymphocytes (0.125 × 10^5^/well to 1 × 10^5^/well, in quadruplicate) were added and incubated at 37 °C and 5% CO_2_ for 24 h. Cells were then discarded, and plates were washed. Antibodies secreted by spleen lymphocytes were detected by adding biotin-conjugated anti-mouse IgG for 2 h followed by extraavidin-peroxidase (Merck) for 1 h. After washing, spots were developed with 3-amino-9-ethylcarbazole (AEC, Merck) and H_2_O_2_ for 15–30 min at room temperature. Finally, the plates were washed with tap water and left to dry in a dark place. The spots were counted by an automatic counter (EliSpot reader AID, Autoimmun Diagnostika GmbH, Strassberg, Germany). Results are expressed as number of spots per 10^6^ cells.

### 2.10. Cell Proliferation

Splenocytes (3 × 10^5^ cells/100 µL) were cultured in quadruplicate to quantify specific proliferation in 96-well U-bottom culture plates (TPP, Tissue Culture Plates, Trasadingen, Switzerland). Cells were stimulated with inactivated HM175-HP (10^5^ TCID_50_/well) or remained non-stimulated for 6 days at 37 °C and 5% CO_2_. To quantify the proliferative activity, a cell proliferation ELISA kit (Roche Diagnostics GmbH, Mannheim, Germany) based on the incorporation of 5-bromo-2′-deoxyuridine (BrdU) was used according to the manufacturer’s instructions. Results are expressed as the ratio between the absorbance of stimulated cells and that of non-stimulated cells (proliferation index) of animals showing an HAV-specific response.

### 2.11. Spleen Memory and Naïve Lymphocytes

The memory and naïve phenotypes of spleen lymphocytes of immunized BALB/c mice were determined by flow cytometry, following the gating strategy schematically represented in Figure S1. For this, lymphocytes were treated with cold PBS-FBS-NaN_3_ (Merck), and cell receptors were blocked with 100 μL of rat serum (1/10 dilution) for 10 min. The Mouse Naïve/Memory T cell ID Panel, with monoclonal antibodies against CD3, CD4, CD8, CD44, CD62L conjugated with different fluorochromes, was used to detect subpopulations of T cells. For intracellular staining, cells were washed, incubated with cell fixation/permeabilization solution (Thermo Fisher Scientific) for 30 min, washed with the permeabilizer, incubated with anti-mouse IFN-γ antibody for 30 min, and washed again. Samples were analyzed with Gallios™ Cytometer (Beckman Coulter) at the Scientific and Technological Centers of the University of Barcelona (CCiT-UB). Data were processed with FlowJo software v.10 (Becton, Dickinson and Company, Franklin Lakes, NJ, USA).

### 2.12. Statistical Analysis

Prism 10 Software (GraphPad Software Inc., Boston, MA, USA) was used for the statistical analysis of the data. The Shapiro–Wilk’s test and Levene’s test were used to assess normality and variance homogeneity, respectively. Homogeneous and normal data were analyzed using one-way ANOVA test followed by Tukey’s post hoc test, whereas nonparametric data were analyzed using Mann–Whitney U test, unless otherwise specified. Results are presented as mean ± standard error of the mean (SEM) for the indicated values, and significant differences were considered when *p* < 0.05.

## 3. Results

### 3.1. HM175-HP and HM175-L0 Have Similar Antigenic Properties

**Figure 1:**
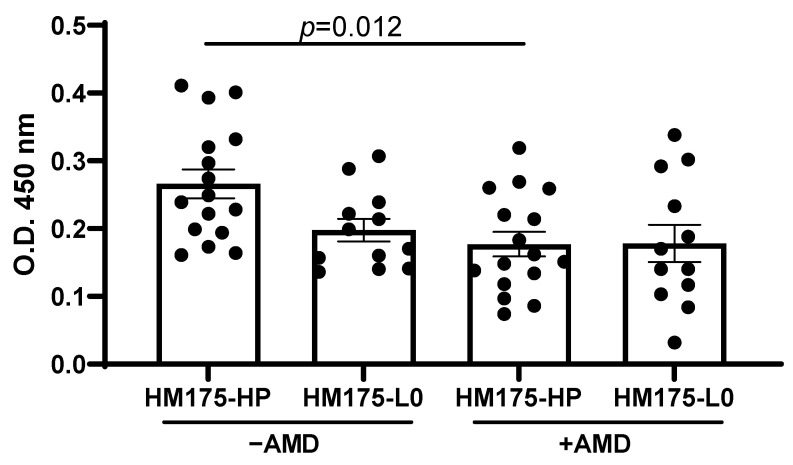
HAV HM175-HP shows similar antigenic properties than HM175-L0. Recognition of viral strains produced in the absence or presence of AMD by anti-HAV human convalescent plasma was assessed by ELISA. Data are shown as the mean ± SEM from 2 independent experiments (n = 16 HM175-HP; n = 12 HM175-L0). The mean O.D. value of the negative controls of each experiment was subtracted prior to combining the data from different experiments. *p*-values were determined by one-way ANOVA with Tukey’s post-test. AMD: Actinomycin D.

To analyze whether the amino acid changes at positions 123 of the VP1 and 134 of the VP2 in the HM175-HP strain alter the antigenic properties of viral capsids, we assessed the recognition of HM175-HP and HM175-L0 viral populations by anti-HAV human convalescent plasma (Figure 1). Because HM175-HP strain was obtained after the adaptation of HM175-L0 strain to grow in the presence of AMD [7], we also assessed the recognition of both viral populations grown in that condition. The viral capsids of HM175-HP and HM175-L0 bound similarly to human convalescent plasma, either in the absence or in the presence of AMD, indicating that both strains share similar antigenic structures. Notably, HM175-HP grown in the absence of AMD showed higher recognition than that of the same virus grown in the presence of AMD.

### 3.2. Humoral Immune Responses Against HM175-HP and HM175-L0 Are More Prominent in BALB/c Mice than in C57BL/6 Mice

To establish the best mouse model for assessing the immunogenic capacity of our candidate HAV vaccine, we compared the immune response induced by HM175-HP vaccine in two mouse strains: C57BL/6 and BALB/c mice.

**Figure 2:**
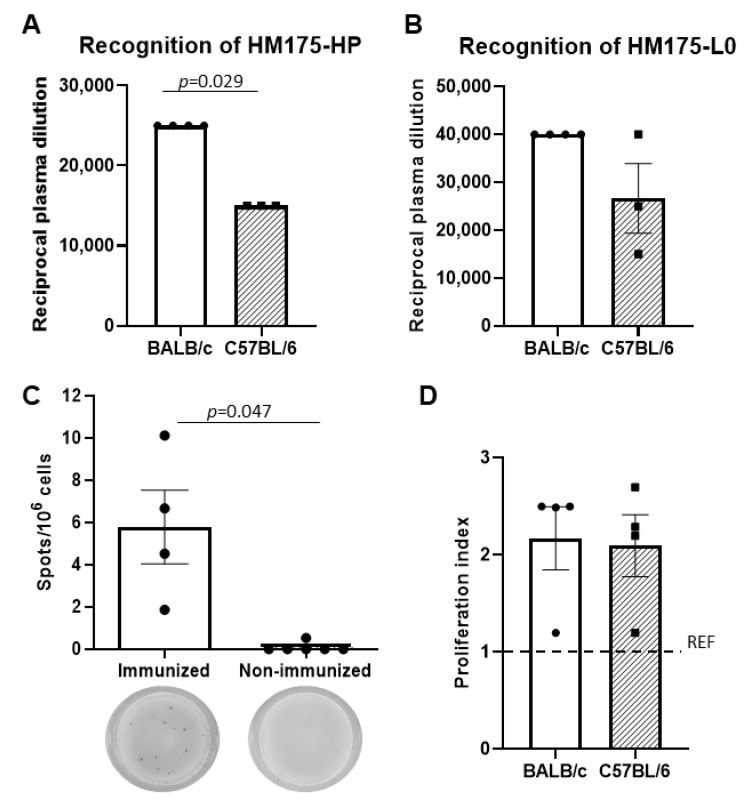
BALB/c mice show higher humoral immune response to HM175-HP than C57BL/6 mice. (**A**) Plasma levels of anti-HAV IgG recognizing the homologous HM175-HP capsid antigen at day 56 since the first immunization. Results are expressed as the mean ± SEM of the reciprocal of the highest plasma dilution providing a positive signal for each animal sample (n = 4). (**B**) Plasma levels of anti-HAV IgG recognizing the heterologous HM175-L0 capsid antigen at day 56 since the first immunization. Results are expressed as in (**A**). (**C**) Counts of anti-HM175-HP antibody-secreting splenocytes from immunized (n = 4) and non-immunized (n = 6) BALB/c mice. Data are shown as the mean ± SEM of spots per 10^6^ cells; representative wells of spots developed against HM175-HP in immunized and non-immunized BALB/c mice are depicted below. (**D**) Proliferation index of splenocytes from BALB/c (n = 4) and C57BL/6 (n = 4) mice under HM175-HP stimulation. Results are expressed as mean ± SEM. Dash line represents the mean value of the REF group (OVA-immunized animals). *p*-values between BALB/c and C57BL/6 mice were analyzed by unpaired two-sided Mann–Whitney test.

Although both mice strains showed plasma anti-HAV IgG levels at day 56 after the first immunization, the response was stronger in BALB/c mice than in C57BL/6 mice, particularly against the homologous HM175-HP capsid antigen (Figure 2A,B).

In addition, the number of anti-HAV antibody-secreting splenocytes from HM175-HP-immunized BALB/c mice ranged between 1.9 × 10^6^ and 1.0 × 10^7^ (5.8 × 10^6^ ± 1.7 × 10^6^) spleen cells and was significantly higher than that in non-immunized animals (Figure 2C).

### 3.3. Cellular Immune Responses Against HM175-HP Are Similar in BALB/c and C57BL/6 Mice

Cellular immune response developed by splenocytes from both C57BL/6 and BALB/c mice strains was established by the proliferative response after HM175-HP stimulation. The proliferation rate was about 2 in both mice strains, with no differences between groups (Figure 2D).

### 3.4. Female BALB/c Mice Produce a Stronger Humoral Immune Response Against HM175-HP and HM175-L0 than Male BALB/c Mice

After concluding that BALB/c mice developed a stronger humoral immune response to HM175-HP and HM175-L0 than C56BL/6 mice, we compared the immune response in female and male BALB/c animals following HM175-HP immunization. The immune response was assessed in terms of antibodies, number of antibody-secreting cells in spleen, and splenocyte proliferation.

**Figure 3:**
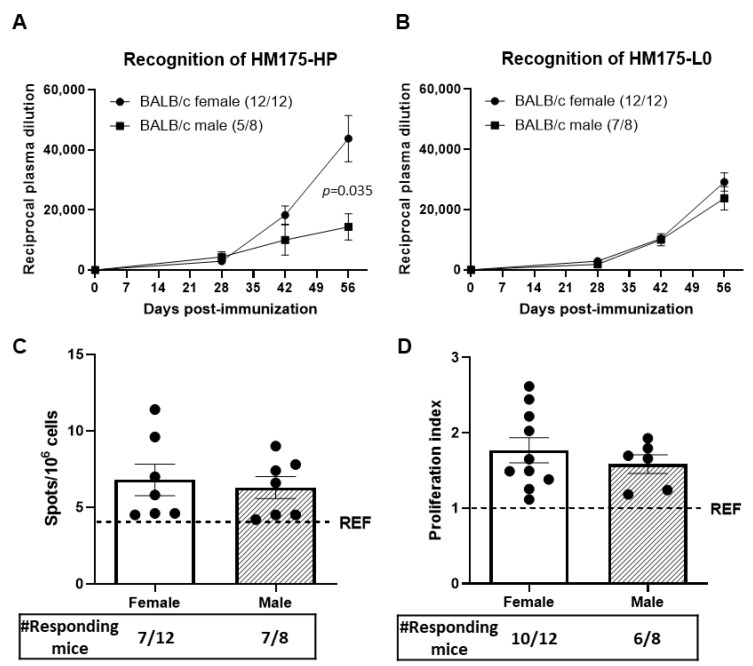
Female BALB/c mice produce a stronger immune response than BALB/c males against HM175-HP and HM175-L0. (**A**) Plasma levels of anti-HAV IgG recognizing the HM175-HP in both female and male mice throughout the immunization process. Results are expressed as the mean ± SEM of the reciprocal of the highest plasma dilution providing a positive signal for each animal sample, considering only those animals showing a positive response. The number of animals showing positive recognition at 56 days since the first immunization, which are indicated in brackets. (**B**) Plasma levels of anti-HAV IgG recognizing the HM175-L0 in both female and male mice throughout the immunization process. Results are expressed as in (**A**). (**C**) Counts of anti-HM175-HP antibody-secreting splenocytes from female and male BALB/c mice. Results are expressed as mean ± SEM of counts in animals that produced a stronger response than REF group, which are indicated below. (**D**) Proliferation index of splenocytes from female and male BALB/c mice under HM175-HP stimulation. Results are expressed as in (**C**). Dash line represents the mean value of the REF group (OVA-immunized animals). *p*-values between female and male BALB/c mice were analyzed by unpaired two-sided Mann–Whitney test.

While all (12/12) BALB/c female mice showed detectable plasma levels of anti-HAV IgG, only 62.5% (5/8) of males presented a positive reaction against HM175-HP virus by ELISA, with anti-HAV IgG levels being significantly higher in BALB/c female mice than in BALB/c male animals at day 56 after the first immunization (*p* = 0.035; Figure 3A). When studying the antibody response against the heterologous HM175-L0 capsid antigen (Figure 3B), all BALB/c female mice (12/12) developed anti-HAV IgG antibodies, whereas the success in BALB/c male mice was 87.5% (7/8).

In addition, no differences due to sex in the number of specific anti-HM175-HP antibody-secreting cells from the spleen of immunized mice were observed, with 6.8 × 10^6^ ± 1.0 × 10^6^ splenocytes in female BALB/c mice and 6.3 × 10^6^ ± 0.7 × 10^6^ splenocytes in male BALB/c mice (Figure 3C). On the other hand, the splenocytes from female and male BALB/c animals showed a similar proliferation index under HM175-HP stimulation (Figure 3D).

### 3.5. Humoral and Cellular Immune Responses in Female BALB/c Mice Immunized with HM175-HP Vaccine Are Similar to Those Immunized with a Prototype-like HAV (HM175-L0) Vaccine

Since females were chosen over males, female BALB/c mice were then vaccinated with either HM175-HP or HM175-L0 vaccines. To compare the immune response induced by both HAV vaccines, we quantified the following: (i) anti-HAV IgG plasma levels against HM175-HP and HM175-L0 elicited by both vaccines during 8 weeks; (ii) the number of spleen anti-HM175-HP antibody-secreting cells at the end of the study; (iii) the cellular proliferative response of spleen cells under HM175-HP stimulation; and (iv) the spleen lymphocyte composition.

#### 3.5.1. Comparison of Anti-HAV IgG Levels and Neutralizing Antibodies Induced by HM175-HP Vaccine and the Prototype-like HAV Vaccine HM175-L0

**Figure 4:**
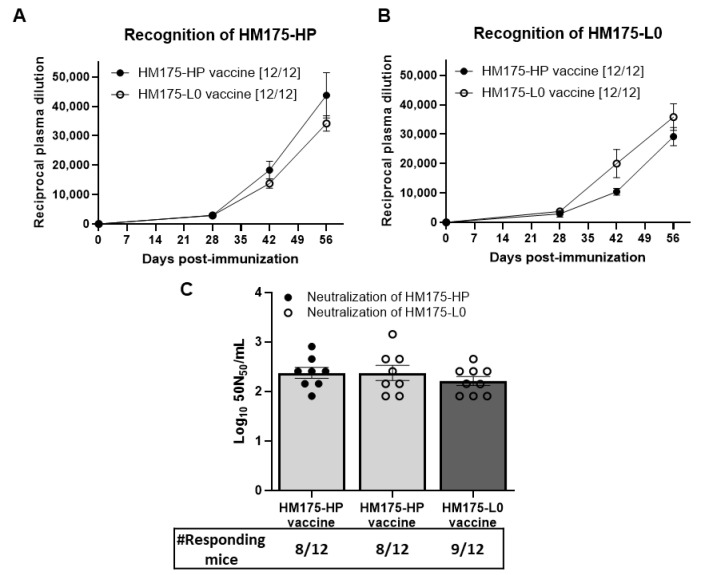
Both HM175-HP and HM175-L0 vaccines induce similar humoral immune responses in female BALB/c mice. (**A**,**B**) Plasma levels of anti-HAV IgG recognizing the HM175-HP (**A**) or the HM175-L0 (**B**) in both HM175-HP and HM175-L0 vaccine groups throughout the immunization process. Results are expressed as the mean ± SEM of the reciprocal of the highest plasma dilution providing a positive signal for each animal sample, considering only those animals showing a positive response. The number of animals showing positive recognition at 56 days since the first immunization is indicated in brackets. (**C**) Neutralizing antibody titers against HM175-HP and HM175-L0 in plasma from immunized mice expressed as the Log_10_ of the number of units per mL of plasma capable of neutralizing 50% of 1 × 10^5^ TCID50 units in 50% of the replicates (Log_10_ 50N50 U/mL). Neutralizing antibody titers in plasma from HM175-HP-immunized mice were tested against both HM175-HP and HM175-L0 strains, while those in plasma from HM175-L0-immunized mice were tested against HM175-L0. The number of mice that developed neutralizing antibody response is indicated below. *p*-values between HM175-HP and HM175-L0 vaccine groups were analyzed by unpaired two-sided Mann–Whitney test.

Both HM175-HP- and HM175-L0-vaccinated groups developed detectable plasma levels of anti-HAV IgG capable of recognizing HM175-HP and HM-175-L0 virus that were already detectable at day 28 after the first immunization and were the highest at day 56 (Figure 4A,B). At day 56, the reciprocal of the highest plasma dilution providing a positive reaction against HM175-HP virus was 43,750 ± 7715 and 34,167 ± 2599 in the HM175-HP- and HM175-L0-vaccinated groups, respectively (Figure 4A), whereas the titers against the HM175-L0 virus were 29,167 ± 3067 and 35,833 ± 4516 in the HM175-HP- and HM175-L0-vaccinated groups, respectively (Figure 4B).

In addition, the neutralization capacity of these antibodies developed by either HM175-HP and HM-175 L0 vaccination was established against HM175-HP and HM175-L0 viruses. Importantly, anti-HAV IgG antibodies induced by HM175-HP vaccination were not only able to recognize HM175-L0 virus but also neutralize it (Figure 4C). In this HM175-HP-vaccinated group, 66.7% (8/12) of the mice presented detectable neutralizing antibody levels against the HM175-L0 and the HM175-HP, with 41.7% (5/12) of the HM175-HP-immunized mice showing detectable neutralizing antibody levels against both HM175-HP and HM175-L0. The neutralization potencies of these HM175-HP-vaccinated mice against HM175-HP and HM175-L0 were 2.4 ± 0.11 and 2.4 ± 0.12 Log_10_ 50N_50_/mL, respectively.

In the HM175-L0-vaccinated group, 75.0% (9/12) of mice presented detectable neutralizing antibody levels against the HM175-L0, with neutralization potencies of 2.2 ± 0.1 Log_10_ 50N_50_/mL (Figure 4C). No significant differences in HM175-L0 neutralization capacity were observed between HM175-HP- and HM175-L0-vaccinated groups.

#### 3.5.2. Comparison of Anti-HAV IgG1 and IgG2a Levels Induced by HM175-HP Vaccine and a Prototype-like HAV Vaccine HM175-L0

**Figure 5:**
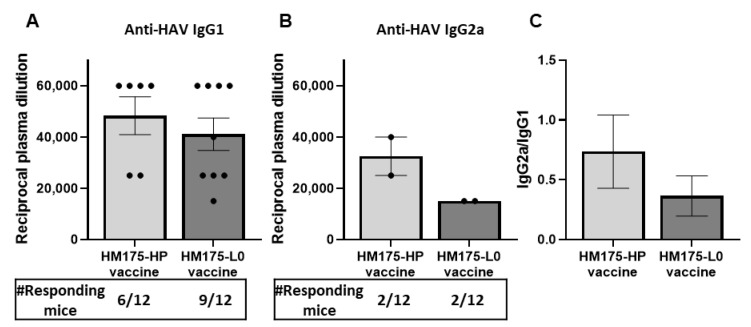
Both HM175-HP and HM175-L0 vaccines induce similar levels of anti-HAV IgG1 and IgG2a in female BALB/c mice. (**A**,**B**) Plasma levels of anti-HAV IgG1 (**A**) or anti-HAV IgG2a (**B**) recognizing the homologous antigens in both HM175-HP and HM175-L0 vaccine groups at 56 days since the first immunization. Results are expressed as the mean ± SEM of the reciprocal of the highest plasma dilution providing a positive signal for each animal sample, considering only those animals showing a positive response; the number of responding mice is indicated below. (**C**) IgG2a/IgG1 ratio calculated as the ratio between the reciprocal of the highest plasma dilution being positive for anti-HAV IgG2a and that for anti-HAV IgG1. *p*-values between HM175-HP and HM175-L0 vaccine groups were analyzed by unpaired two-sided Mann–Whitney test.

To further evaluate the immune response generated by both HM175-HP and HM175-L0 vaccines, the plasma titers of anti-HAV IgG1 and IgG2a subclasses were measured at day 56 after the first immunization (Figure 5). In mice, IgG1 is typically associated with Th2-type immune responses, while IgG2a is linked to Th1-type immune responses [16]. Concerning IgG1, both vaccinated groups showed similar titers of anti-HAV IgG1 antibodies, with values of 48,333 ± 7379 and 41,111 ± 6334 in the HM175-HP- and HM175-L0-vaccinated groups, respectively (Figure 5A). In addition, 16.7% (2/12) of the animals in each group developed anti-HAV IgG2a antibodies, with the titer of anti-HAV IgG2a being higher in the HM175-HP-vaccinated group (32,500 ± 7500) than in the HM175-L0-vaccinated group (15,000 ± 0) (Figure 5B). Although the statistical significance was not achieved due to the small number of BALB/c females developing anti-HAV IgG2a antibodies, the IgG2a/IgG1 ratio was consistent with a trend towards a higher IgG2a response in the HM175-HP-vaccinated group (Figure 5C).

#### 3.5.3. Comparison of the Number of Antibody-Secreting Cells to HM175-HP Virus Induced by HM175-HP Vaccine and the Prototype-like HAV Vaccine HM175-L0

**Figure 6:**
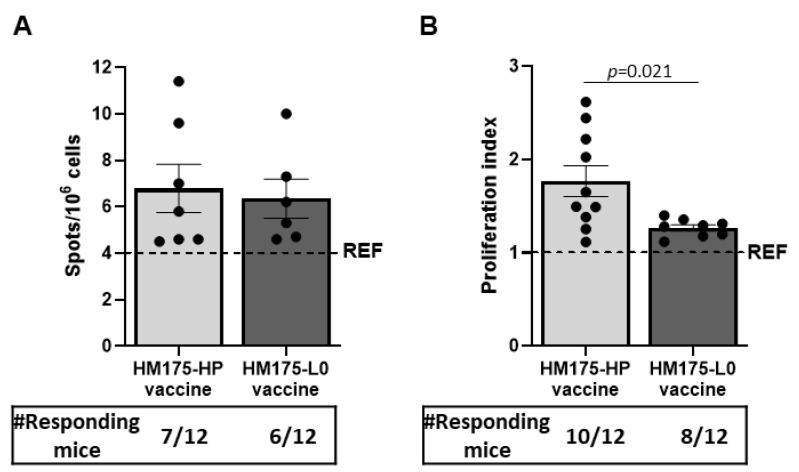
The HM175-HP vaccine induces similar counts of antibody-secreting splenocytes but higher cellular immune responses than the prototype-like HAV vaccine. (**A**) Counts of anti-HM175-HP antibody-secreting splenocytes from HM175-HP- and HM175-L0-vaccinated female BALB/c mice. (**B**) Proliferation index of splenocytes obtained at day 56 from HM175-HP- and HM175-L0-vaccinated female BALB/c mice and stimulated with inactivated HM175-HP. Dash line represents the mean value of the REF group (OVA-immunized animals). Results are expressed as mean ± SEM of animals in each group that produced a stronger response than REF group; the number of these responding mice is indicated below. *p*-values between HM175-HP- and HM175-L0-immunized BALB/c mice were analyzed by unpaired two-sided Mann–Whitney test.

The number of anti-HM175-HP-virus-secreting cells ranged between 4.5 and 11.4 per 10^6^ (6.8 × 10^6^ ± 1.0 × 10^6^) cells in HM175-HP-vaccinated animals and between 4.6 and 10 per 10^6^ (6.4 × 10^6^ ± 0.8 × 10^6^) cells in mice with HM175-L0 vaccine (Figure 6A). No significant difference was found between vaccinated groups, meaning that both vaccines stimulated a similar number of B cells in the spleen at day 56 after the first immunization.

#### 3.5.4. Comparison of the Cellular Immune Responses Induced by HM175-HP Vaccine and the Prototype-like HAV Vaccine HM175-L0

After 8 weeks since the first immunization, the splenocytes of both HM175-HP- and HM175-L0-vaccinated groups proliferated in response to HM175-HP stimulus (Figure 6B). Animals in the HM175-HP vaccine group showed higher proliferation index than those in the HM175-L0 vaccine group. In addition, 83.3% (10/12) of the mice in the HM175-HP vaccine group showed an increase in splenocyte proliferation following the stimulus, while in the HM175-L0 group, 66.6% (8/12) of the mice showed such a response.

**Figure 7:**
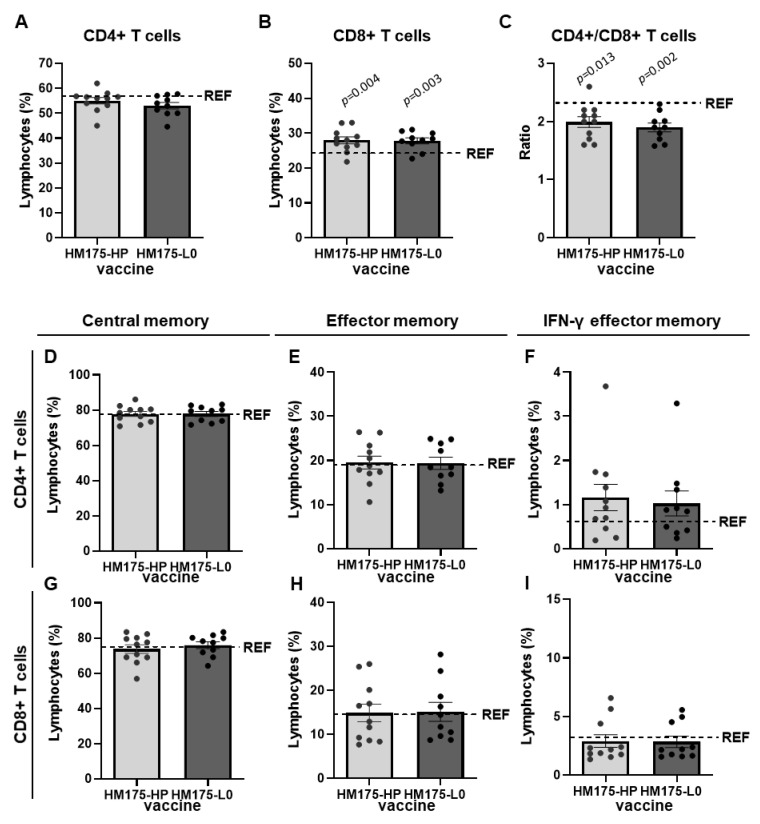
Comparison of spleen lymphocytes subsets in HM175-HP- or HM175-L0-immunized mice. (**A**,**B**) Proportion of CD4+ T cells (**A**) and CD8+ T cells (**B**) among spleen lymphocytes. (**C**) CD4+/CD8+ T cell ratio. (**D**–**I**) Proportion of central memory (**D**), effector memory (**E**), and IFN-γ effector memory (**F**) subsets among CD4+ spleen lymphocytes. Proportion of central memory (**G**), effector memory (**H**), and IFN-γ effector memory (**I**) subsets among CD8+ spleen lymphocytes. Dash line represents the mean value of the REF group (OVA-immunized animals). *p*-values between HM175-HP- or HM175-L0-immunized mice and REF group were analyzed by unpaired two-sided Mann–Whitney test.

To further assess the cellular immune response, isolated spleen lymphocytes obtained at day 56 from the first immunization were phenotypically characterized (Figure 7 and Figure S1). In the absence of HAV stimulus, we observed that administration of HM175-HP and HM175-L0 vaccines resulted in similar proportions of CD4+ T cells than the REF group (Figure 7A). However, when considering the percentage of CD8+ T cells, values in both HAV-vaccinated groups did not differ but were significantly higher than those observed in the REF group (Figure 7B). Therefore, the CD4+/CD8+ ratio in both vaccinated groups were lower than that in the REF animals (Figure 7C).

For both CD4+ and CD8+ T lymphocytes, the proportion of central memory cells (CMC, CD44 + CD62L+), effector memory cells (EMC, CD44 + CD62L), and IFN-γ+ cells within EMC (EMC IFN-γ+) was determined. Although there was a slight increase in EMC IFN-γ+ CD4+ cells in both vaccinated groups compared to the REF group, no statistical significance was achieved. Similarly, no significant changes were observed in the other CD4+ and all the CD8+ T cell subsets between HM175-HP-vaccinated and HM175-L0-vaccinated groups and between these groups and the reference one (Figure 7D–I).

## 4. Discussion

Current HAV vaccines are very effective and provide life-long protection. However, they are based on cell culture-adapted HAV strains that replicate poorly, which results in costly and time-consuming procedures. Consequently, mass vaccination programs in lower-middle-income countries with increasing incidence of clinically important HAV infections are unaffordable, and vaccine shortages often hinder the control of large outbreaks that frequently occur in high-income countries. A novel HAV vaccine based on a fast-growing strain capable of producing higher antigen yields in shorter times [13], such as the HM175-HP clone, would therefore constitute a great advantage, allowing mass vaccination of children and avoiding vaccine shortages. Here, we demonstrate in a preclinical approach that this fast HM175-HP clone induces a similar immunization when compared to the HM175-L0 clone, a prototype-like HAV vaccine.

The codon usage modifications acquired by the HM175-HP strain during its adaptation to AMD-induced cellular shut-off, along with two amino acid replacements in the viral capsid, were initially associated with changes in recognition by monoclonal antibodies [10]. In the present study, despite the lower recognition of HM175-HP grown in the presence of AMD, no differences were observed regarding the recognition by an anti-HAV convalescent plasma between the parental HM175-L0 and the HM175-HP (Figure 1), indicating that the latter has similar antigenic properties than that of the HM175-L0. In addition, these results provided further evidence that the HM175-HP-based vaccine could be produced in the absence of AMD, favoring the capsid folding that better resembles that of the HM175-L0, which resembles one of the prototypes of the current vaccines capable of inducing a protective response against natural HAV infection.

To assess the immunogenicity of our candidate vaccine, a preliminary study was conducted using two mouse strains, BALB/c and C57BL/6, that were immunized with inactivated HM-175-HP virus. Both mouse strains developed anti-HAV antibodies that recognized both HM175-HP and HM175-L0 viruses. Moreover, we found that spleen lymphocytes from both BALB/c and C57BL/6 mice strains proliferated against HM175-HP virus, and BALB/c mice (C57BL/6 mice were not tested) showed spleen anti-HM175-HP antibody-secreting cells. Although no differences were observed regarding the cellular proliferative response under HM175-HP stimulation between BALB/c and C57BL/6 mice, the BALB/c group showed higher anti-HAV antibody levels than the C57BL/6 group when considering the HM175-HP virus (Figure 2). These results are consistent with previous studies reporting that BALB/c mice tend to produce a stronger humoral response than C57BL/6 mice [17,18,19,20]. In addition, it has been reported that the protective antibody responses in BALB/c mice are higher than that in C57BL/6J mice in the case of a vaccination with a *Plasmodium* protein [21].

Because sexual dimorphism in immune response has been reported [22,23], we also compared the response in terms of anti-HAV IgG levels, anti-HAV antibody-secreting cells and cellular proliferative response between male and female BALB/c mice vaccinated with HMP-175 HP (Figure 3). In accordance with previous studies reporting that post-pubertal females have higher immunoglobulin levels [23], female BALB/c mice showed higher anti-HMP175-HP antibody levels and higher seroconversion rates (100% vs. 62.5%) than BALB/c males. Thus, further validation of our vaccine candidate was performed on adult female BALB/c mice.

To validate HP175-HP virus as a vaccine candidate, we compared the results obtained after HP175-HP vaccination with those obtained after HM175-L0 vaccination, a prototype-like HAV vaccine. Firstly, both HM175-L0 and HP175-HP vaccines, formulated with alum, induced the production of anti-HAV antibodies, already perceived on day 28, i.e., after two doses of the vaccine, and increasing until day 56, the last day of the study. Importantly, both vaccines induced antibodies against their autologous virus strain and against the heterologous virus strain, meaning that those animals vaccinated with the HP175-HP, the fast-growing HAV strain, had antibodies that recognize the HP175-L0, the strain used in one of the current HAV vaccines (Figure 4B). It is worth noting that specific anti-HAV IgG antibodies are a major mechanism of protection, with 10–20 mIU/mL being the cut-off for seroprotection [24,25], levels that are usually achieved with HAV vaccine [24]. Moreover, passive immunization with human immunoglobulin is used for pre- and post-exposure prophylaxis against HAV infection [26]. It must be emphasized that IgG-mediated protection relies, at least in part, on the neutralizing activity of anti-HAV antibodies [24,26]. In this regard, it is important to note that we found that plasma from HM175-HP-immunized mice was able to neutralize the HM175-L0 strain (Figure 4C), and would therefore be expected to neutralize the wild-type virus as HM175-based vaccine-induced antibodies do.

Despite both HM175-L0 and HP175-HP vaccines inducing the production of anti-HAV antibodies in all immunized mice, we found detectable levels of neutralizing antibodies in 8–9 out of 12 vaccinated animals. The discrepancies between total anti-HAV antibodies and neutralizing anti-HAV antibodies can be related to the use of cell culture-based assays to assess neutralizing antibody levels. These assays are based on the decrease in infectious titer, attributable to antibodies that block the entry of viruses into the host cells; they fail, however, in detecting non-neutralizing antibodies that can also confer some protection. Indeed, non-neutralizing antibodies have shown protection comparable to that of neutralizing antibodies in humanized mouse models of SARS-CoV-2 [27].

In agreement with the results of plasma antibodies, we detected splenic plasma cells producing HM175-HP antibodies on day 56 after the first immunization, which were similar in both vaccinations (Figure 6A), demonstrating the ability to produce such specific antibodies at such a time.

On the other hand, both vaccinated groups produced anti-HM175-HP IgG1 antibodies (Figure 5A), which are associated with Th2 immune response [16]. This response can be due to the adjuvant we used, alum, with recognized Th2 response stimulation [28,29]. However, the vaccination with HM175-HP virus showed a tendency to produce a greater IgG2a response than that elicited by HM175-L0 vaccination (Figure 5B), suggesting that this vaccination also elicits a higher Th1 response, which is essential for host defense against intracellular pathogens [30]. Further studies focused on the analysis of cytokines released by splenocytes under virus stimulation will clarify these results.

Previous studies have demonstrated that HAV vaccines induce HAV-specific proliferative T cell responses [5]. In this study, we assessed the cellular immune response induced by HM175-HP and HM-175-L0 vaccines by establishing cellular proliferation against HM175-HP virus. The levels of cell proliferation were similar in both vaccine groups, and the higher rate of success in the HM175-HP vaccine group compared to the HM175-L0 group (83.3% vs. 66.6%) could be due to the homologous stimulus (Figure 6B). As stated before, the elicitation of a cellular immune response is important to fight against intracellular viruses [30]. These results were consistent with the spleen T cell composition after vaccination. For all considered cell subsets, no differences appeared between our vaccine candidate HM175-HP and the prototype-like HM175-L0 vaccine. Both HM175-HP- and HM175-L0-vaccinated groups showed higher proportions of CD8+ T cells (and lower CD4+/CD8+ T cell ratio) than those found in the reference animals (Figure 7B,C). These could indicate the activation of cytotoxic T cells, which are involved in the cellular immune response because they are able to recognize antigens on the surface of infected cells and induce their apoptosis [31]. In fact, it has been reported that transgenic mice infected by HAV and protected with a peptide vaccine increased virus-specific CD8+ T cell frequencies in the liver, which is associated with a reduced viral RNA abundance and a lower liver injury [32].

We found no significant alterations in CMC and EMC after vaccination with respect to reference animals (Figure 7D–I). CMC is derived from naïve T cells after activation and subsequently differentiate into T effector cells [33,34]. Our results partially agree with those reported with HAV vaccine in healthy humans [35], in which HAV vaccination is associated with higher CMC frequencies but unchanged EMC frequencies, which are increased after pathogen exposure as a primary response to contribute to the viral clearance [35]. On the other hand, we observed no significant changes in the EMC IFN-γ+ subsets when compared to the proportions in reference animals. Although IFN-γ secretion after in vitro stimulation has been reported in other virus immunizations [36], our results referred to unstimulated cells. However, it is worth noting that virus-vaccinated mice showed a tendency to increase the proportion of EMC CD4+ IFN-γ+. This is interesting because it could reflect the activation of the Th1 immune response, involved in the reaction against intracellular pathogens such as HAV. Further studies should focus on the secretion of IFN-γ and other Th1 cytokines after in vitro stimulation.

The use of mouse models incapable of recapitulating HAV infection is a limitation of this study. Moreover, mouse models often fail to replicate human immune responses, which reinforces the need for caution when extrapolating mouse data to human contexts. However, the efficacy of the HM175-based vaccine in humans is well established. In this study, we have compared the efficacy of our vaccine candidate HM175-HP with that of the prototype-like HM175-L0 in the mouse model. The lack of differences between the immune responses induced by both vaccines suggests that our candidate could be a good alternative for developing a cost-effective vaccine against HAV.

## 5. Conclusions

In this study, we demonstrate that both HM175-HP and HM175-L0 strains have similar antigenic properties and elicit a similar immune response. These findings indicate that our fast-growing HM175-HP strain shows potential to help in the development of a new HAV vaccine, reducing the costly and time-consuming procedures of the currently available vaccines against hepatitis A. Further studies are needed to explore the potential of the HM175-HP vaccine in clinical settings.

## Data Availability

The raw data supporting the conclusions of this article will be made available by the authors on request.

## Supplementary Materials

The following supporting information can be downloaded at: https://www.mdpi.com/article/10.3390/vaccines13050446/s1, Figure S1: Representative image of spleen cell analysis procedure.
