# Supplementary material for: Immunogenic Properties of a Novel Hepatitis A Vaccine Candidate Based on a Fast-Growing Viral Strain
Source: Vaccines (Basel). 2025 Apr 23;13(5):446. doi: 10.3390/vaccines13050446 (PMC12115964; doi:10.3390/vaccines13050446)
Supplement: Supplementary file 1 [file vaccines-13-00446-s001.zip › vaccines-3575332-supplementary.pdf]

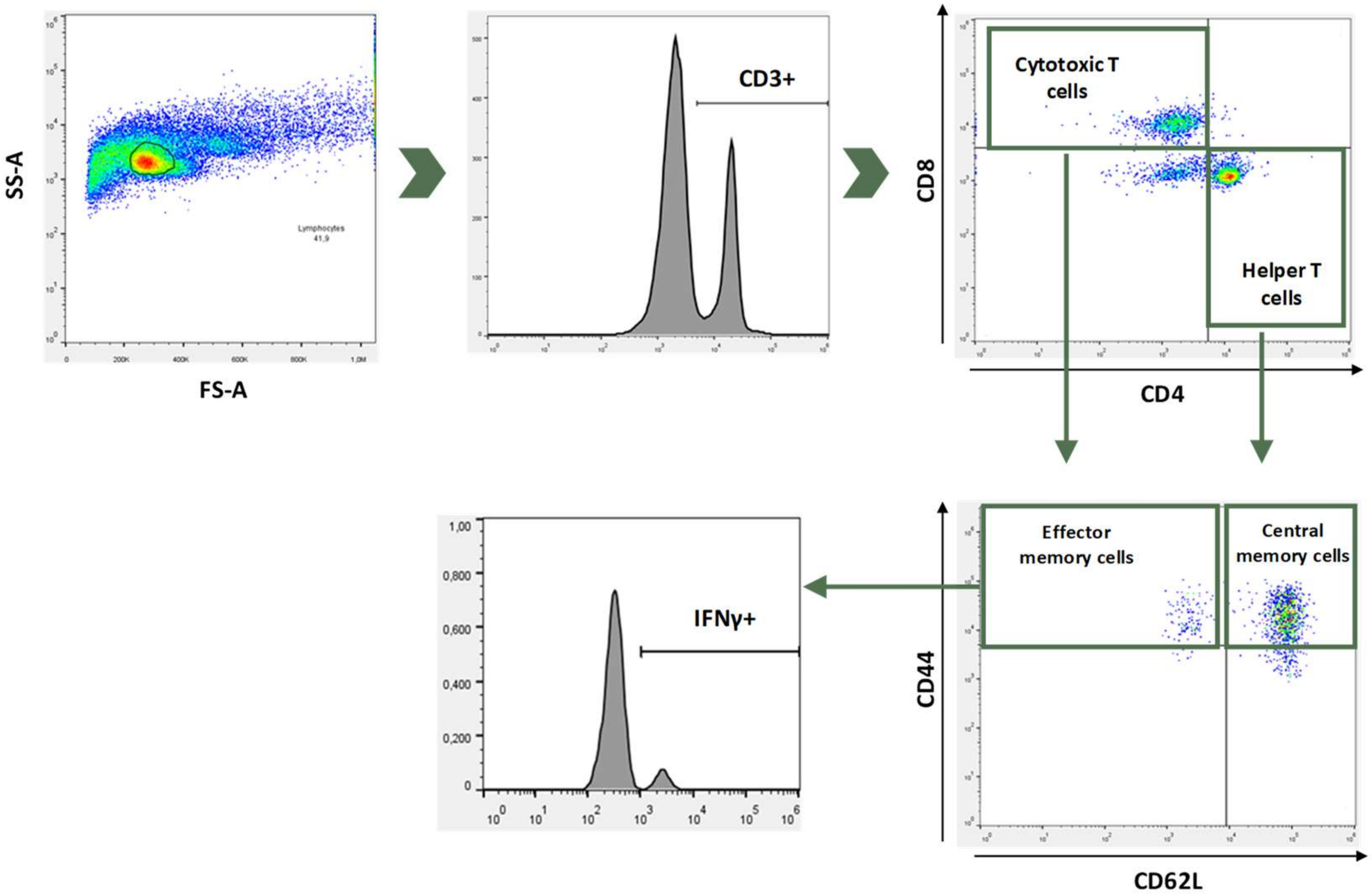

Figure S1. Representative image of spleen cell analysis procedure. Side scatter (SS-A) and forward scatter (FS-A) cytogram was used to gate lymphocytes. Then, CD3+ cells were selected and further classified into CD4+ and CD8+ T cells. Both populations were then analysed according to the expression of CD44 and CD62L molecules, which allows the identification of effector memory cells (EMC, CD44+CD62L-) and central memory cells (CMC, CD44+CD62L+). Finally, IFN-γ expression was analysed in the effector memory cell subsets.
